# Customizing GPT for natural language dialogue interface in database access

**DOI:** 10.1186/s44342-024-00020-5

**Published:** 2024-11-01

**Authors:** Jin-Dong Kim, Kousaku Okubo

**Affiliations:** 1 Database Center for Life Science (DBCLS), Joint Support-Center for Data Science Research (ROIS-DS), Research Organization of Information and Systems (ROIS), 178-4-4 Wakashiba, Kashiwa, 277-0871 Chiba Japan; 2 National Institute of Genetics (NIG), 1111 Yata, Mishima, 411-8540 Shizuoka Japan

**Keywords:** Natural language dialogue interface, Large language models, ChatGPT, GPTS, Anatomy, Database

## Abstract

The paper presents *Anatomy3DExplorer*, a customized ChatGPT designed as a natural language dialogue interface for exploring 3D models of anatomical structures. It illustrates the significant potential of large language models (LLMs) as user-friendly interfaces for database access. Furthermore, it showcases the seamless integration of LLMs and database APIs, within the GPTS framework, offering a promising and straightforward approach.

## Introduction

Databases serve as vital resources particularly for scientific endeavors. However, accessing them frequently requires proficiency in specialized languages such as *SQL* or *SPARQL*. While some web services offer tailored interfaces for specific databases, becoming accustomed to those custom interfaces also often involves a learning curve. Meanwhile, *natural language dialogue interfaces (NLDIs)* are expected to serve as the ultimate user-friendly interface for accessing databases, leveraging users’ innate familiarity with natural language, and thus fundamentally eliminating the need for learning a new interface. Consequently, numerous efforts have been made to develop NLDIs for enhanced database accessibility. Since the emergence of *large language models (LLMs)*, which has profoundly reshaped the landscape of research and development, they have shown a great potential to offer an ideal interface for database access. Indeed, numerous attempts have been made to implement such interfaces [1, 2]. However, most of these efforts have centered around *text-to-SQL* or *text-SPARQL* research, which still remains largely within the domain of ongoing research.

In our study, we direct our attention to the conversational capabilities of LLMs, specifically focusing on *ChatGPT* [3, 4], which has demonstrated remarkable effectiveness as a conversational agent. Notably, *OpenAI* has recently introduced customized versions of ChatGPT, known as *GPTs* [5], which we view as a significant opportunity to explore. As a proof of concept, we developed a customized GPT, named *Anatomy3DExplorer*, specifically tailored for accessing the  *BodyParts3D anatomy database* [6]. During our use of the interface over *BodyParts3D*, we found this approach is promising. In this paper, we present the results (Section 2) and describe the methods employed in our study (Section 3).

## Results

*Anatomy3DExplorer* is a customized ChatGPT developed to serve as an NLDI for *BodyParts3D*^1^, a database of human anatomical structures in which shapes and positions of human body parts are represented by 3D models [6]. Anatomy3DExplorer is available from the GPT store^2^.

**Fig. 1:**
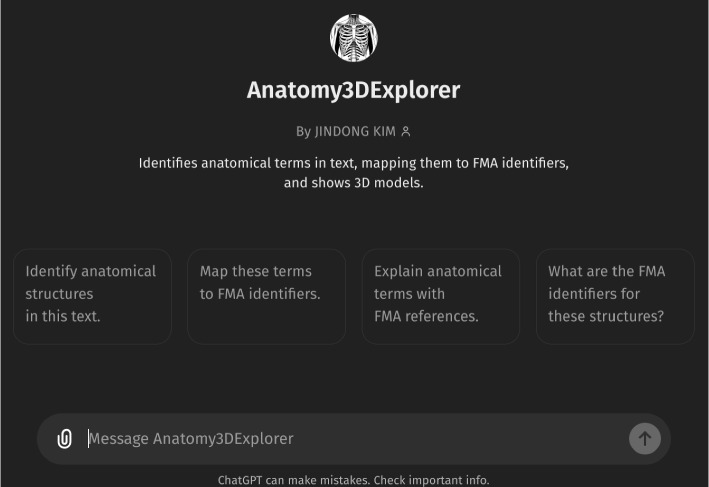
The initial look of *Anatomy3DExplorer*

**Fig. 2:**
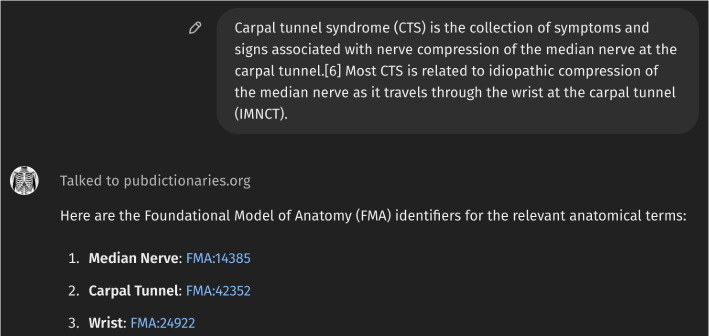
Response of *Anatomy3DExplorer* when a block of text is provided. The text is a copy from the page of *Carpal tunnel syndrome (CTS)* of *Wikipedia*

**Fig. 3:**
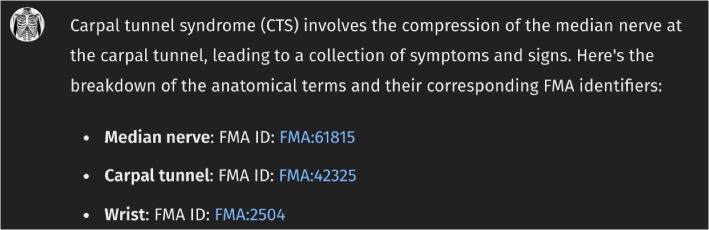
Response of *ChatGPT* with hallucinated identifier references in the absence of consulting *PubDictionaries*

Figure 1 presents the initial interface of *Anatomy3DExplorer*, where it is described as a tool designed to identify anatomical terms in text, map them to corresponding FMA identifiers, and display them as 3D models. Figure 2 illustrates a response from *Anatomy3DExplorer* when provided with a block of text. As depicted in the figure and indicated in the initial screen, anatomical terms found in the provided text are shown, and they are accompanied by their respective FMA identifiers. Notably, the message “*Talked to pubdictionaries.org*” signifies an essential customization integrated into *Anatomy3DExplorer* to address the issue of halluciation associate with identifier references. It is a well known issue that LLMs are prone to generating hallucinations, such as false ontology identifiers, which poses a significant challenge when utilizing them for database interfaces. Figure 3 shows a response of GPT to the same input, but without consulting to *PubDictionaries*. While it shows FMA identifiers, in fact, they are all incorrect, exemplifying a common case of hallucination. To address this issue, *Anatomy3DExplorer* integrates an external dictionary lookup service, *PubDictionaries*, for retrieving FMA identifiers. *PubDictionaries* is a public repository of dictionaries, offering dictionary lookup service upon registration of a dictionary [7]. As the dictionary lookup process is deterministic, concerns regarding hallucinations are effectively mitigated.

**Fig. 4:**
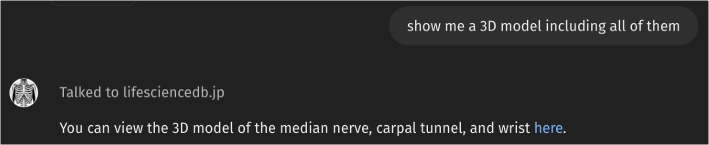
Example of instructing *Anatomy3DExplorer* to create a 3D model of anatomy structures

**Fig. 5:**
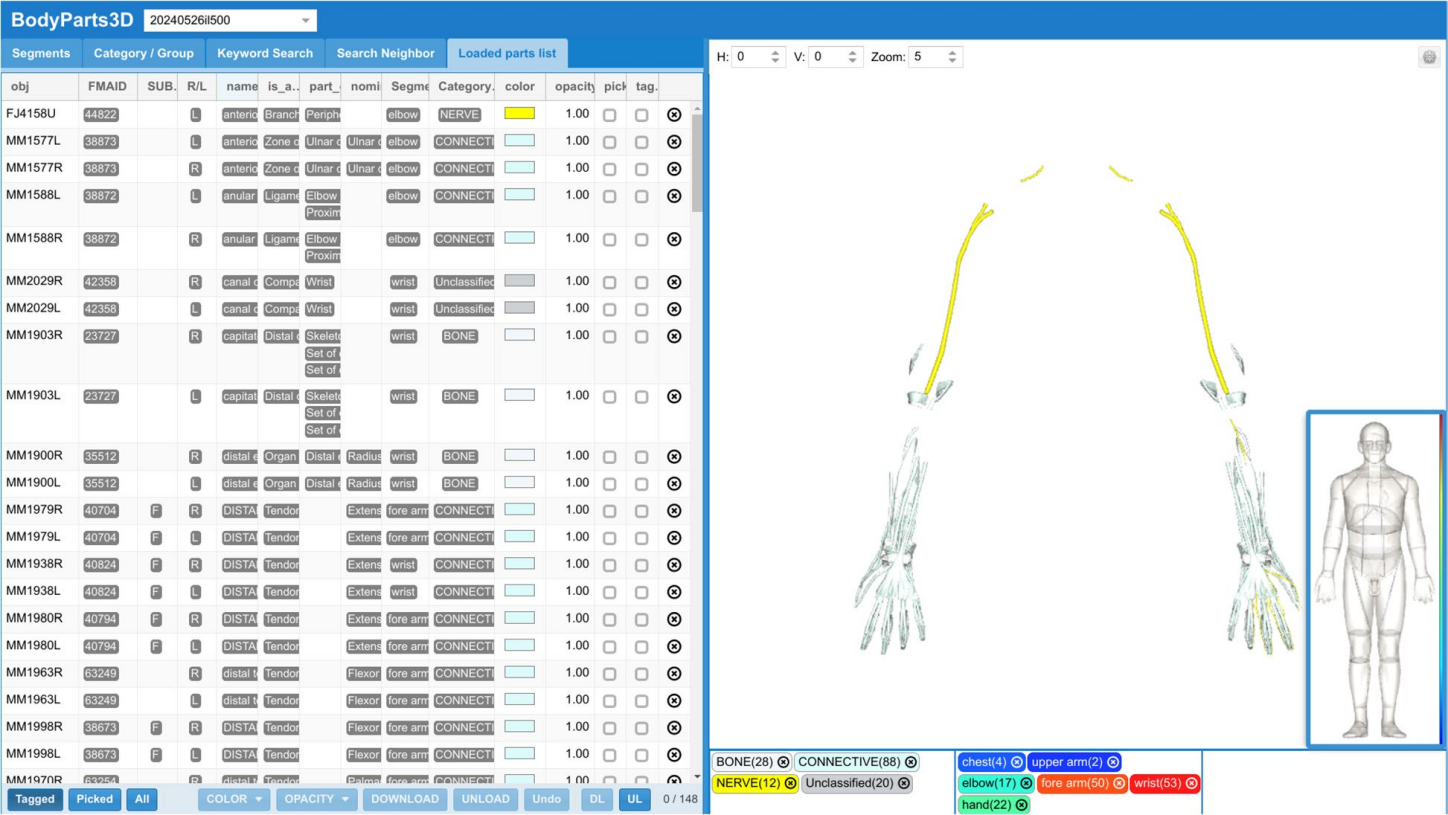
An interactive 3D model generated by *BodyParts3D* in response to a user request delivered through the chat interface of *Anatomy3DExplorer*

Figure 4 illustrates how *Anatomy3DExplorer* responds when prompted to display a 3D model encompassing all the anatomical terms identified thus far. The message "*Talked to lifesciencedb.jp*" signifies another customization made to *Anatomy3DExplorer*: instead of having GPT generate a 3D model, it communicates with another external service, *BodyParts3D*, hosted at lifesciencedb.jp. Since BodyParts3D generates an interactive 3D model that cannot be displayed within the chat interface, *Anatomy3DExplore*r provides a link that directs the user to an external page displaying the 3D model of relevant anatomical structures for c*arpal tunnel syndrome* (Fig. 5).

**Fig. 6:**
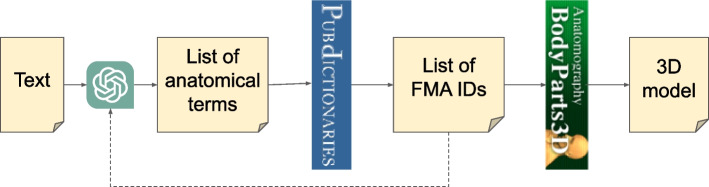
A typical workflow with *Anatomy3DExplorer*

Figure 6 presents a diagram illustrating the workflow demonstrated in the example dialogue up to this point. When presented with a block of text, *Anatomy3DExplorer* utilizes the capabilities of *ChatGPT* and *PubDictionaries* to generate a list of anatomical terms alongside their corresponding FMA identifiers. Ultimately, the workflow can be concluded with the creation of a 3D model of the anatomical structures, facilitated by *BodyParts3D*.

### Managing the unpredictability of LLM outputs

**Fig. 7:**
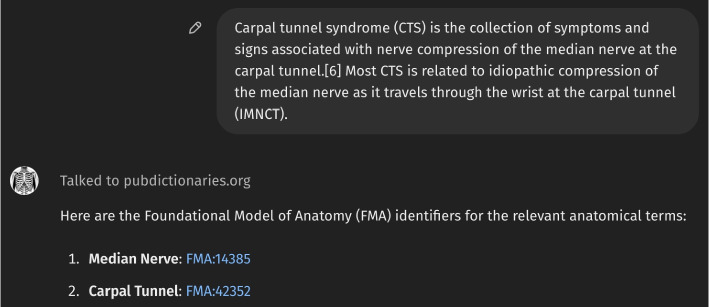
Example of instructing to *Anatomy3DExplorer* to improve information

**Fig. 8:**
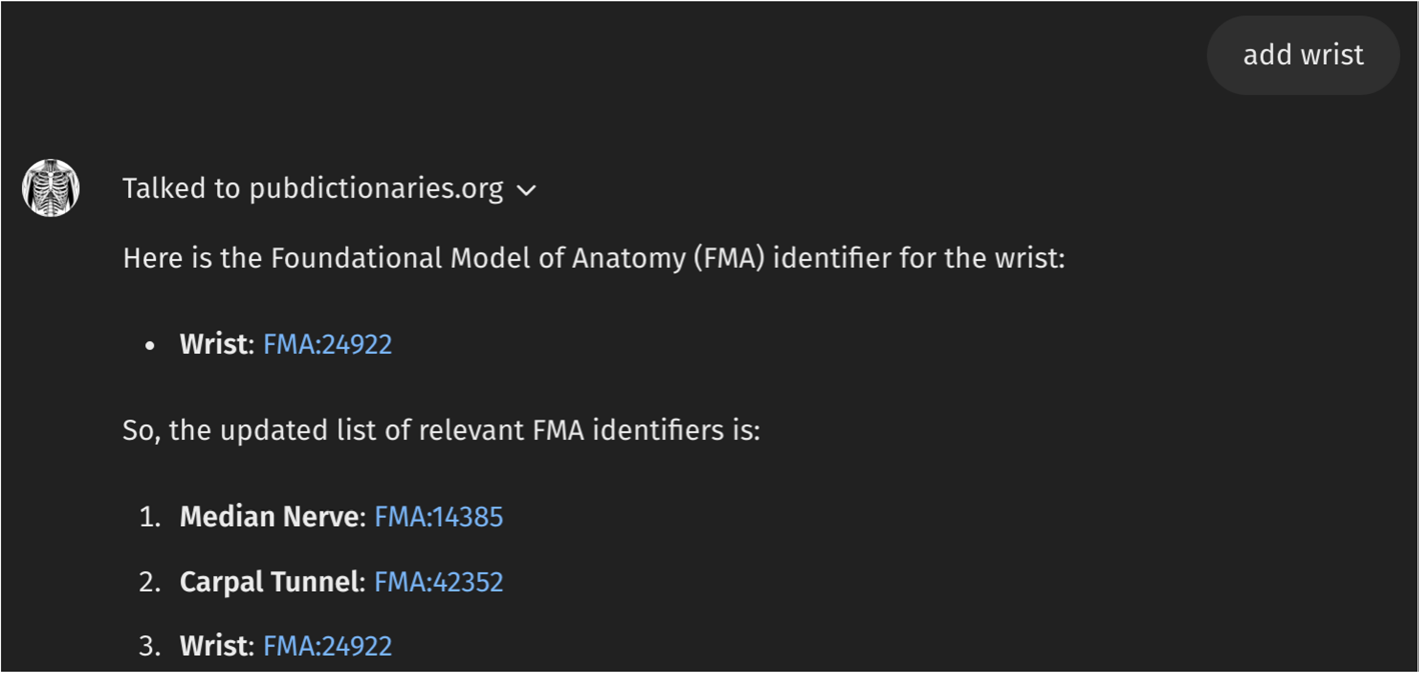
Example of instructing to *Anatomy3DExplorer* to improve information

It is widely recognized that the outputs of LLMs are inherently unpredictable. To demonstrate this, we conducted multiple tests of *Anatomy3DExplorer* using identical inputs. Figure 7 presents a distinct output from Fig. 2, notably missing the term “*wrist*” as an anatomical reference. Likewise, the output of LLMs is unpredictable and occasionally may yield imperfect responses. However, we have found that a notable strength lies in the interactive nature of LLMs. Users can engage in ongoing communication with LLMs to refine and enhance the outputs. Figure 8 provides an illustrative example: a user identifies the absence of “*wrist*” as problematic, requests its addition, and the adjustment is promptly made. This adjustment can be almost effortlessly accomplished by instructing LLMs in natural language. As a result, the response now includes a comprehensive list of anatomy terms alongside their corresponding FMA identifiers. Once again, *PubDictionaries* is consulted to retrieve the correct FMA identifier of for the term “*wrist*.”

In Fig. 6, the dotted line denotes the possibility of iterative cycles involving human intervention.

## Methods

As a customized ChatGPT, *Anatomy3DExplorer* incorporates two actions for communicating with two external services: *BodyParts3D* [6] and *PubDictionaries* [7]. *BodyParts3D* offers an API that enables the retrieval of 3D models of anatomical structures by providing it with a list of FMA identifiers, as demonstrated below:

/FMASearch_SegmentUI/latest/?id=FMA14385&id=FMA42352&id=FMA24922&expansion=all

The example path provided above generates a 3D model containing all the anatomical structures related to the *median nerve* (FMA14385), *carpal tunnel* (FMA42352), and *wrist* (FMA24922), as illustrated in Fig. 5. Given its convenience in obtaining 3D models of anatomy, an “*action*” to communicate with this API has been seamlessly integrated into *Anatomy3DExplorer*. This is achieved by instructing *Anatomy3DExplorer* with the OpenAPI schema of the API. See Appendix A for the detail of the schema.

However, utilizing the API requires users to furnish the FMA identifiers themselves, which can be a daunting task. It is important to note that this challenge is not exclusive to *BodyParts3D* but rather a prevalent issue across many databases. While we could request ChatGPT to fetch the FMA identifiers, ChatGPT and other large language models (LLMs) are notoriously unsuitable for this type of task (See Fig. 3). This is where *PubDictionaries* proves effective. While *PubDictionaries* hosts numerous dictionaries, *Anatomy3DExplorer* specifically utilizes the FMA dictionary to retrieve accurate FMA identifiers of interest during dialogues with users. See Appendix B for the detail of the schema for *PubDictionaries*.

## Discussion and conclusion

In this study, we demonstrated the potential of customizing ChatGPT, referred to as GPTs, as a simple yet powerful approach to developing a user-friendly natural language dialogue interface (NLDI) for database access. The primary outcome of the current work, *Anatomy3DExplorer*, is available to the public through the GPT store.

The key contributions of this work can be broadly summarized as:

1. Customizing ChatGPT to function as a natural language dialogue interface for a human anatomy database, and
2. Effectively mitigating hallucination issues related to retrieving accurate identifiers and biomedical images.

While *Anatomy3DExplorer* provided a proof-of-concept application with FMA identifiers and *BodyParts3D*, the same technique is transferable to other scenarios. For instance, GPT can be customized to accurately retrieve *Human Phenotype Ontology (HPO)* identifiers to access the *Mouse Genome Informatics (MGI)* database. MGI is a comprehensive database of mouse genetics, genomics, and biology, facilitating the study of human health and disease. This database can be accessed through various ontologies, including HPO. A user of the customized GPT might issue prompt such as:

I want to access the MGI page about decreased head circumference

The customized GPT would then retrieve the correct HPO identifier, *HP:0040195* for *Decreased head circumference*, and provide the URL for the relevant page:

https://www.informatics.jax.org/diseasePortal?termID=HP:0040195

This streamlined process significantly simplifies access for the user.

This approach can be extended to further customize GPT for retrieving MedGen identifiers and accessing MedGen pages. A user could then issue a prompt like:

I want to access the MedGen page about the same phenotype

The customized GPT would retrieve the MedGen identifier for *Decreased head circumference* (*473122*) and provide the URL for the corresponding MedGen page:

https://www.ncbi.nlm.nih.gov/medgen/473122

This unified, user-friendly interface provides a convenient platform for researching phenotypes across multiple databases. Although it is yet a hypothetical scenario, the proof-of-concept system presented in this work demonstrates that such a scenario can be implemented using the same technique. We plan to explore this direction further in future work.

## Data Availability

No datasets were generated or analyzed during the current study.

## Footnotes

1 https://lifesciencedb.jp/bp3d/

2 https://chatgpt.com/g/g-jJ69Vqn6F-anatomy3dexplorer

## Code availability

Code (the OpenAPI schema) is included in the appendix.
